# Cognitive Functions and Humor in Anxiety Disorders: A Virtual Reality Approach

**DOI:** 10.1192/j.eurpsy.2026.11351

**Published:** 2026-07-31

**Authors:** S. Mammarella, L. Giusti, D. De Luca, A. Salza, R. Roncone

**Affiliations:** 1 University of L’Aquila; 2 University Unit Rehabilitation Treatment, Early Interventions in mental health, Hospital S. Salvatore, L’Aquila, Italy

## Abstract

**Introduction:**

Virtual reality (VR) represents an innovative opportunity for the assessment of cognitive functions, thanks to its ability to create immersive and standardized environments (Miskowiak et al., J Psychiatr Res. 2022; 95: 27–30), allowing evaluations to be conducted in safe and controlled settings.

**Objectives:**

This exploratory study aims to investigate the relationships between sense of humor, loneliness, optimism and cognitive performance (attention, vigilance, working memory, switching, inhibition, and perseveration) as measured in a VR environment.

**Methods:**

The sample consisted of 40 participants, including 11 patients from the U.O.S.D. Early Rehabilitation Interventions - TRIP, ASL 1 Abruzzo, diagnosed with Generalized Anxiety Disorder, and 21 university students (control group). An observational case-control study design was adopted. The following instruments were administered: the **Multidimensional Sense of Humor Scale** (MSHS, 24 items) to assess humor, the **UCLA Loneliness Scale** (10 items) to evaluate perceived loneliness, and the **Jalowiec Coping Scale – Optimistic Coping subscale** (9 items) to assess coping strategies. Additionally, **Nesplora Aquarium (Giunti Psychometrics)**, a virtual reality-based test, was employed to evaluate executive functions.

**Results:**

No statistically significant differences in humor were observed between the two groups, whereas differences in cognitive functions were detected. The sense of humor was positively correlated with better inhibitory control (r = .325; p = .024), indicating lower impulsivity and fewer related errors. Additionally, overall humor scale scores were positively associated with optimism (r = .338; p = .038). A negative attitude toward humor was correlated with reduced switching ability (r = –.323; p = .048), reflecting lower cognitive flexibility, while humor production was linked to decreased sustained attention.

**Conclusions:**

Our data suggests that, in the assessment of cognitive abilities in young people with a biopsychosocial vulnerability, it is essential to consider how executive functions may influence the sense of humor, a key factor for well-being and quality of life, and therefore warrants further investigation.

**Disclosure of Interest:**

None Declared

